# The Staff at Gravesend Hospital

**Published:** 1914-05-23

**Authors:** 


### The Staff at Gravesend Hospital.

A UNIQUE OCCASION.

At the monthly meeting of the Committee of Management of the Gravesend Hospital held on Monday, at which the President, the Earl of Darnley, presided, a very interesting and unique presentation took place. His Lordship was supported by Mr. H. D. Stephenson (Chairman of the Hospital Committee), Mr. T. W. J. Byrne, J.P., Mr. J. H. Cooper, jun., Mr. C. H. Golding-Bird, Alderman H. E. Davis, C.C., Mr. C. E. Dismorr, Dr. Firth, Mr. Arthur Fowle, the Rev. Canon E. L. Gedge, B.A., Mr. J. D. Hartley, Dr. Lawrence, Mr. H. Edwin May, Mr. C. J. Pinching, Mr. H. E. Porter, Mr. C. E. Robbs, Mr. A. Wilson Rose, Colonel J. H. Sankey, J.P., Mr. C. Snelling, Mr. Geo. Wood, J.P., and Mr. W. Pearson, secretary of the hospital.

After the ordinary business of the meeting had been dispensed with, Lord Darnley said: "I am very pleased to be able to be here to-day to take some share in offering, on behalf of the hospital, some recognition of the lengthy and valuable services rendered by Mr. Pinching and Dr. Firth—thirty-six and thirty-one years respectively. I believe this is an additionally interesting occasion, as it happens to be the birthday of both these gentlemen, and I am sure you will wish me to convey your very heartiest good wishes and the sincerest hopes that they may have many happy birthdays in the future. It is a fact that in the yearly reports of hospitals with which I have been acquainted a paragraph has invariably appeared that the committee thank the honorary medical staff, but we cannot sufficiently acknowledge the work gratuitously given by the honorary staff. It has been my lot in some work I have had to undertake to hear a certain number of hard things said about the doctors in connection with their work among the poorer classes; now it is my very firm belief that there is no class of the whole community that has given so much voluntary help, both in their private practice and in their services to the hospitals, as the doctors. We very much hope that these small mementoes will remind Mr. Pinching and Dr. Firth, of the great gratitude of the committee of management, and also of their thanks for the interest they have shown in the poor patients. They appreciate most highly their work, and also their valuable help in matters of administration, and I may tell you that the hospital intends to commemorate their services in a very worthy and suitable manner by calling two of the wards after your names. I have the very greatest pleasure, on behalf of the hospital management, in asking your acceptance of these inkstands as a means of showing in some slight way our heartfelt gratitude."

Dr. Firth, in acknowledgment, said: "When I came into this room to-day I came in with the feeling of a patient going to the operating theatre. I must thank you for these presents in acknowledgment of the little service we have done, and I am sure I thank you for the kind words and for the way in which you have shown your appreciation of my work and also that of my colleague, Mr. Pinching, who is unavoidably absent to-day. It is over thirty years since I was connected with the hospital, and I think Mr. Pinching's connection is longer still, and I can only assure you that it is with great regret and sorrow that we leave you now. Time has been very lenient with both our lives, and our energies are still unimpaired and our spirits still strong to do any work. We recognise, however, that we must bow to the rule stating the retiring age, made some time back—and a very wise one. In looking back over years gone by on the numerous changes and the enormous developments which the hospital has undergone, I should like to say that Gravesend not only owes thanks to the medical staff, but also to the committee of management, because by their wide views and their skilful management of the hospital they have kept the same up to date and abreast with modern science. These inkstands, which we shall highly prize, will always remain with us as mementoes of the Gravesend Hospital, and we shall look back on the times we have spent together within the walls of the hospital as times which have not been spent in vain.

Lord Darnley stated that he wished to emphasise the fact that Mr. Pinching had served thirty-six years, and Dr. Firth thirty-one, and he thought it a very remarkable record indeed. He believed he was right in saying that Mr. Pinching and his family have been connected with the work of the hospital since the absolute commencement.

Portraits of both gentlemen have been hung in the board-room, on the mounts of which is set forth the date of their appointment on the staff.

